# Effect of Electrochemical Aryl Diazonium Salt Modification on Interfacial Properties of CF/PEEK Composites

**DOI:** 10.3390/ma17122899

**Published:** 2024-06-13

**Authors:** Mingchen Sun, Xuekuan Li, Hansong Liu, Chengyu Huang, Kai Wang, Yan Zhao

**Affiliations:** School of Materials Science and Engineering, Beihang University, Beijing 100191, China; smc703@126.com (M.S.); lxk1771@163.com (X.L.); liuhansongzhfc@foxmail.com (H.L.); huangchengyu@buaa.edu.cn (C.H.); wangkai@buaa.edu.cn (K.W.)

**Keywords:** carbon fiber, PEEK, interface, aryl diazonium salt modification, carbon nanotube

## Abstract

The interfacial properties between carbon fiber (CF) and thermoplastic resin are relatively weak, which can be problematic for composites in structural applications. Improving the surface roughness of CF is regarded as an effective way to enhance the interface of composites. However, most CF modifying methods are complex and time-consuming, which cannot meet the demand for industrial production. Therefore, it is of great significance to research a fast technique of CF surface modification to strengthen the interface of composites. Herein, a one-pot reaction based on the aryl diazonium salt modification was applied to enhance the interface between CF and poly ether ether ketone (PEEK) resin. Carbon nanotubes (CNTs) were linked to CF by p-phenylenediamine (PPD) via cyclic voltammetry (CV). The surface morphology, chemical characteristics and surface energy of modified CF illustrated the effectiveness of this method, and the interfacial properties of as-prepared modified CF/PEEK demonstrated the increased tendency. All the CF was treated within 5 min and the interfacial shear strength (IFSS) of CF/PEEK was increased to the maximum of 99.62 MPa by aryl diazonium salt modification. This work may shed some light on the industrialized application of CF reinforced high-performance engineering thermoplastic composites.

## 1. Introduction

Continuous carbon-fiber-reinforced high-performance thermoplastic composites (CFRTP) uses continuous carbon fiber (CF) as the reinforcement and high-performance thermoplastic polymer materials such as poly ether ether ketone (PEEK), poly phenylene sulfide (PPS), and polyetherimide (PEI) as the matrix. Compared with traditional continuous carbon-fiber-reinforced thermosetting composite (CFRTS) materials, CFRTP materials have unique advantages such as strong impact resistance, high damage tolerance, low moisture absorption, corrosion resistance, short forming cycle, and the ability to be processed twice. Among the high-performance thermoplastic matrices, PEEK has better heat resistance and mechanical properties which has been widely used in aerospace, energy, transportation, biomedical and other industries [1].

Interfacial performance is worth noting during the use of composite materials. Good interfacial bonding is conducive to stress transfer between resin and fiber, which can give full play to the advantages of composite materials. On the contrary, a weaker interface will be more likely to cause interfacial debonding during the loading process of the composite material, leading to material failure. However, during the preparation process of CFRTP, due to the high viscosity, poor wettability, and chemically inert surfaces, it is difficult to form chemical bonds and other interactions between thermoplastic resin and fibers. Therefore, the interfacial properties of thermoplastic composite materials are poor and need to be further improved.

Main theories to improve interface strength include chemical bonding theory and mechanical interlocking theory. Among them, the chemical bonding theory believes that the adhesion of the resin to the fiber can be improved by the chemical bond between fiber and resin, thereby enhancing the strength of the interface. As a result, researchers have proposed a variety of CF surface treatment methods, such as acid treatment [2], surface oxidation [3], etc., and also studied the modification of PEEK matrix [4]. The mechanical interlocking theory believes that increasing the roughness of the fiber surface can enhance the mechanical interlocking between the fiber and the resin when the resin is well impregnated, thereby improving the interface strength. The methods to achieve mechanical engagement include polymer growth or nanoparticle modification. Among them, attaching nanoparticles to the surface of CF is the most common way. Wang et al. [5] theoretically proved that chemical grafting of carbon nanotubes (CNTs) on the surface of CF can enhance the interface. Sun et al. used electrodeposition and electropolymerization to deposit CNTs on the CF surface to enhance the interfacial strength of CF/epoxy(CF/EP) composites [6]. Jiang et al. [7] used electrochemical oxidation treatment to graft oxygen-containing functional groups on the surface of CF and deposited graphene oxide(GO) to enhance the interfacial strength of CF/EP composites. Wen et al. [8] used electropolymerization to react on the CF surface to generate diacetone acrylamide (DAAM), acrylic acid (AA) and phenol (phenol). In addition, some researchers have used surface sizing to play both roles at the same time, treating the fiber surface by synthesizing a sizing agent containing nanoparticles [9,10,11,12].

However, the improvement of interface performance not only needs to consider the feasibility in principle, but also the feasibility and efficiency issues in actual processing. The methods above could significantly improve the interfacial performance, but it takes a long time to achieve modification. Many methods are not suitable for mass production. In some methods of attaching nanoparticles, since there is no interaction between nanoparticles and CF, the attached nanoparticles are easy to fall off, thus failing to achieve the desired modification effect during the storage, transportation and processing of CF.

Some modification methods can achieve continuous and rapid modification, such as plasma irradiation [13], electrochemical treatment [14], etc. However, these methods will damage the structure of CF or sacrifice the surface carbon elements, causing a certain degree of loss to the tensile strength of CF.

The reaction principle of aryl diazonium salt modification is to react the aryl amino group with an oxidant in the electrolyte to generate a diazo group, and then remove the diazo group through a reduction reaction that generate N_2_, generating an aryl free radical, which reacts with substrate bonding to enable the grafting. This method can supply electrons externally and does not directly consume elements on the surface of the substrate for reaction, so it will cause less damage to the CF. In recent years, aryldiazonium salt modification has been used by more researchers in interfacial modification. Common aryl diazo reaction systems include NaNO_2_ as the oxidant and low-temperature reaction systems. Some systems use RNO_2_ (such as dinitroisoamyl ester) at high temperatures to react in solutions or molten urea and other systems. Yan et al. [15] used t-Bu-ONO to graft p-phenylenediamine onto the surface of desizing CF under high temperature and matched it with modified sPEEK and tested the interfacial properties. Beggs et al. [16] modified CF/EP composite materials under the conditions of tBuONO, DCB, and heating. The interfacial shear strength (IFSS) of CF/EP increased by 47%. Delamar et al. [17] tried to use NBu_4_BF_4_ to graft p-nitroaniline onto CF in ACN solvent and reduced it with hypophosphorous acid. The IFSS was increased from 71 MPa to 116 MPa. Cao et al. grafted trifluoromethylbenzene on CF under low temperature and NaNO_2_ environment to improve oil pressure resistance and oleophobicity. 

In addition to these methods, the electrochemical reaction method has the simplest conditions, fast reaction times, and the potential for continuous production. It can be tried as a method of surface modification of CF. Cougnon et al. [18] introduced the principle of electrochemical diazonium salt reduction reaction. Servinis et al. [19] used cyclic voltammetry (CV)-assisted methods to graft p-nitroaniline, p-aminobenzoic acid, and p-nitrobenzoic acid onto the surface of CF through NOBF_4_ salts, and tested the IFSS changes of CF/EP composite. Among them, the effect of grafted amino groups was much higher on carboxyl and amide groups. Servinis et al. [20] also used the same process to graft long-chain hydrophilic groups on the surface of CF. Eyckens et al. [21] also used electrochemical aryl diazonium reaction to graft a benzenesulfonic acid polymer on the surface, making the surface of the carbon fiber appear blue. Andideh et al. [22] used CV to graft HBPU-OH polyhydroxyl functional groups on the surface of CF, and converted the hydroxyl groups into amino groups through chemical means to study the improvement of its interfacial properties. Han et al. [23] used electrochemical methods to graft p-aminophenyl phosphate onto a glassy carbon electrode in a buffer solution to catalyze the decomposition of hydrogen peroxide. Seck et al. [24] used cyclic voltammetry to graft carboxyl, thiol, and amino functional groups on the copper electrode to change the hydrophilicity of the electrode surface and the adsorption performance of heavy metal ions. The above experiments confirmed the feasibility of electrochemical aryl diazonium reaction.

Another problem in the diazo reaction process is that there are certain side reactions. Not only does the connection between the substrate but also the aryl amino group occur on the modified surface, there is also the benzene ring on the grafted molecule as a site to continue to react with the arylamino group. A polymer layer would be formed in this process. Pinson et al. [25] found that during the grafting process through diazonium salt reduction reaction, small molecules will continue to self-polymerize to form a polymer layer.

It is easier to graft small molecules using aryl diazo modification, but it is difficult for small molecules to interact with the PEEK matrix. Therefore, trying to achieve the grafting of rigid nanoparticles on the surface of CF through this reaction is an idea to further improve the interfacial performance of CF/PEEK composites. For example, Oweis et al. [26] used p-phenylenediamine as the grafting bridging agent on the surface of CoCr alloy. In the environment of NaNO_2_ and HCl, they used H_3_PO_2_ as the reducing agent to graft SDS and BP to the alloy surface to form a polymer layer. A good two-step reaction could be achieved by controlling the reaction time. Liu et al. [27] used cyclodextrin protection method to graft CNTs on the surface of CF to increase the surface roughness of CF. The above method provides a reference for applying nanoparticles to the CF/PEEK interface.

In response to the above problems, this article intends to develop a modification method that is fast, efficient, and has the potential for continuous CF surface modification. It uses electrochemical aryldiazonium salt reaction to first connect p-phenylenediamine(PPD) to the CF surface, and then use the same reaction conditions to graft CNTs to the surface of CF to achieve rapid modification of the CF/PEEK interface and study the impact of the modification method on the strength of CF single fiber. On the basis of stabilizing the process, the interfacial properties of CF/PEEK composite materials can be further improved by changing parameters such as reactant type and reactant concentration. This method can match the continuous production of carbon fibers and has the potential to achieve continuous modification of the interface of CF/PEEK composite materials.

## 2. Materials and Methods

### 2.1. Materials

The CF tow (T700-SC-12K) was purchased from Toray Corporation of Tokyo, Japan. The PEEK film was made by Institute of metal research, Chinese academy of science. MWCNT dispersion (TNWDM-M8, 10 wt%) was purchased by Time-Nano, Beijing, China. p-Phenylenediamine (99%, AR), Sulfanilic acid (ASA, 99%, AR) 4-aminobenzoic acid (ABA, 99%, AR) and NaNO_2_ (99%, AR) was purchased from Macklin, Shanghai, China. Hydrochloric acid (HCl, 36%, AR) and Acetone was purchased from Beijing Chemical Works, Beijing, China. 

### 2.2. Preparation

#### 2.2.1. Preparation of Working Electrode 

CF was first desized by using Soxhlet extraction at 120 °C for 6 h to completely dissolve the sizing agent on the surface of CF. Then, the desized CF was dried in an air-circulating oven at 100 °C for 1 h. Take about 7 cm of the pulped CF bundle, fix both ends with copper foil tape, tighten it and stick it on the glass slide. Cover the surface of the copper foil tape with a layer of polyimide (PI) tape to reduce the impact on the conductivity of the electrode.

#### 2.2.2. Preparation of Electrolyte Solution

Component A: 3.93 g of concentrated hydrochloric acid was added to 200 mL deionized water to prepare 0.2 mol/L dilute hydrochloric acid solution. PPD was then added and heated at 50 °C for 10 min to completely dissolve to prepare 10, 20, 30, 40 mmol/L PPD solution. When preparing electrolyte with CNT, 0.2 g CNT dispersion was added to 200 mL PPD solution with a certain concentration. The mixture was stirred evenly with and then sonicated in an ultrasonic instrument for 30 min. All solutions were sealed and refrigerated at 4 °C.

Component B: NaNO_2_ was added into 200 mL deionized water to prepare 10, 20, 30, 40 mmol/L NaNO_2_ solution. The NaNO_2_ solution was sealed and refrigerated at 4 °C.

When conducting the test, 100 mL component A and 100 mL component B were mixed in a 250 mL wide-mouthed bottle. The mixed solution was placed it in an ice-water bath and stirred continuously to react for 5 min at 0–5 °C. If the mixed solution turned reddish-brown, it indicated that the temperature control is not appropriate and a diazonium salt coupling reaction has occurred, resulting in the formation of colored azo-based substances.

#### 2.2.3. Electrochemical Aryl Diazonium Salt Modification of CF

**Figure 1:**
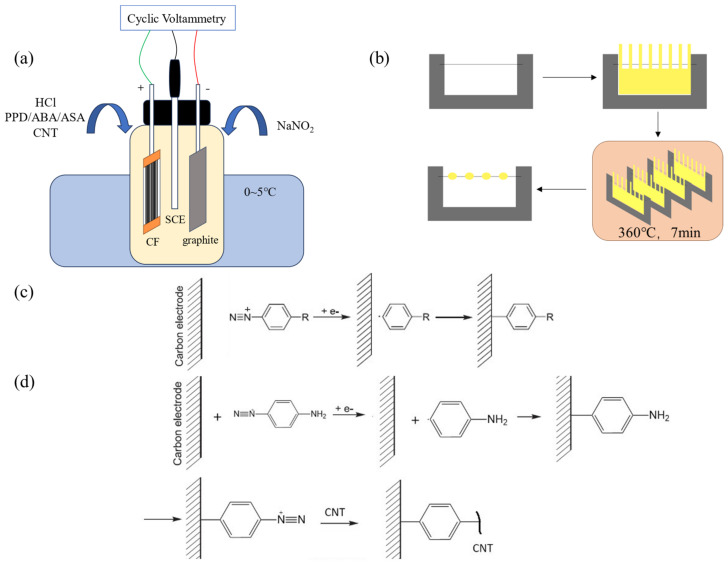
Diagram of sample preparation process (**a**) The process of cyclic-voltammetry-modified CF (**b**) The preparation of micro-bonding samples (**c**) Reaction equation of molecule modifying CF (**d**) Reaction equation of CNTs modifying CF.

The modification process is shown in Figure 1a. The prepared CF working electrode, high-purity graphite plate, and saturated calomel electrode (SCE) were, respectively, connected to the working electrode, counter electrode, and reference electrode terminals of the electrochemical workstation (Shanghai Chenhua CHI660E, Shanghai, China). The test mode was set as Cyclic Voltammetry (CV), and the initial potential, highest potential, lowest potential, and end potential was set in sequence to 0 V (for PPD grafting, the potential is 0.1 V), 0 V, −0.8 V, and 0 V. The number of cycles was 10, the scanning speed was 0.1 V/s and the sampling accuracy was 1 × 10^−3^ A. The modified CF was placed into deionized water and sonicated for 5 min to remove electrolyte and unreacted CNT attached on CF.

### 2.3. IFSS Test

A single fiber of CF was fixed on the C-shaped steel sheet. Then, a piece of PEEK cast film was cut into a comb shape and fixed on the outside of the single fiber with PI tape. The whole sample was heated in the infrared oven at 360 °C for 7 min to obtain a CF/PEEK micro-debonding sample. The diagram of the sample preparation process can be seen in Figure 1b.

The IFSS test was conducted by using the micro-bonding method. The IFSS of each sample was calculated according to Equation (1).

(1) $$\tau =\frac{F}{\pi dl}$$

where *τ* is the IFSS of each sample, *F* is the maximum force during deboning, *d* is the diameter of CF, which equals to 7 μm in this work, and *l* is the length of each micro droplet embedded on the CF.

### 2.4. Physicochemical Characterizations of CFs

The surface morphology of CF was observed by scanning electron microscope (SEM, JEOL JSM-7500, Tokyo, Japan) to determine the sizing removed from the surface, the fiber surface morphology before and after modification and the degree of self-polymerization reactions. The acceleration voltage and beam current were set as 3 kV and 10 nA. The X-ray photoelectron spectrometer analyzer (XPS, ESCALab 220i-XL, ThermoFisher Company, Waltham, MA, USA) was used to test the chemical elements and functional groups on the surface of CF. Al K (1456.6 eV) was used as the radiation source. Surface roughness was tested using an atomic force microscope (AFM, Bruker Dimension icon, Karlsruhe, Germany). We set the scanning range to 3 μm × 3 μm and the scan frequency to 1 Hz. The roughness calculation was performed using NanoScope Analysis 1.7 software. The calculated surface was smoothed twice and the roughness value could be read.

### 2.5. Single Fiber Tensile Test

The single fiber tensile test was conducted on a mechanical testing machine (Instron5967, INSTRON Corporation, Norwood, MA, USA) equipped with a 5N sensor. The sample was prepared in accordance with the standard ASTM D3379 “Testing Method for Strength and Young’s Modulus of High Modulus Monofilament Tensile Materials” [28]. A total of 40 or more single fiber samples were tested for each type of CF to guarantee the data accuracy.

The strength of single fibers were fitted by Weibull distribution. The fitting formula is shown in Equation (2):

(2) $$P=1-exp\left[-{\left(\frac{\sigma }{\sigma_{0}}\right)}^{m}\right]$$

where *P* is the cumulative probability of failure of a CF at applied tensile strengths, *m* is the Weibull modulus or shape parameter of the CF and *σ*_0_ is the Weibull scale parameter or characteristic stress. *P* is determined for each point using Equation (3):

(3) $$P=\frac{i-0.5}{n}$$

where *n* is the number of sample points and *i* is the rank. Equation (2) could be rearranged to a linear form to obtain m and *σ*_0_, which is shown in Equation (4).

(4) $$ln\left[-ln\left(1-P\right)\right]=mln\sigma -mln\sigma_{0}$$

The average breaking strength of CF could be calculated by using Equation (5):

(5) $$\bar{\sigma }=\sigma_{0}\Gamma \left(1+\frac{1}{m}\right)$$

### 2.6. Contact Angle and Surface Energy

The contact angle and surface energy were tested using a dynamic contact angle tester (dataphysics DCAT25). A single experiment used 3 carbon fiber monofilaments, adjusted the immersion depth to 3.5 mm, and the immersion speed to 0.1 mm/s. The immersed liquid were chosen as water and ethylene glycol.

Surface energy was calculated using the OWYK method [29]. The contact angle in different liquids can be calculated by deriving the following Equation (6):

(6) $$\gamma_{l}\left(1+cos\theta \right)=2\left(\sqrt{\gamma_{l}^{p}\cdot \gamma_{s}^{p}}+\sqrt{\gamma_{l}^{d}\cdot \gamma_{s}^{d}}\right)$$

where *θ* is the contact angle, $\gamma_{l}$, $\gamma_{l}^{p}$ and $\gamma_{l}^{d}$ are surface tension and its polar and dispersive component of the liquid which is used to test *θ*. In this work, these three parameters are set as the number in Table 1. $\gamma_{s}^{p}$ and $\gamma_{s}^{d}$ are polarity and disperse component of the testing surface, which could be calculated by linear fitting the data of at least two types of liquids. Total surface energy $\gamma_{s}$ could be calculated by adding $\gamma_{s}^{p}$ and $\gamma_{s}^{d}$.

## 3. Results and Discussion

### 3.1. Effect of the Concentration on Aryl-Diazonium-Salt-Modified CF

**Figure 2:**
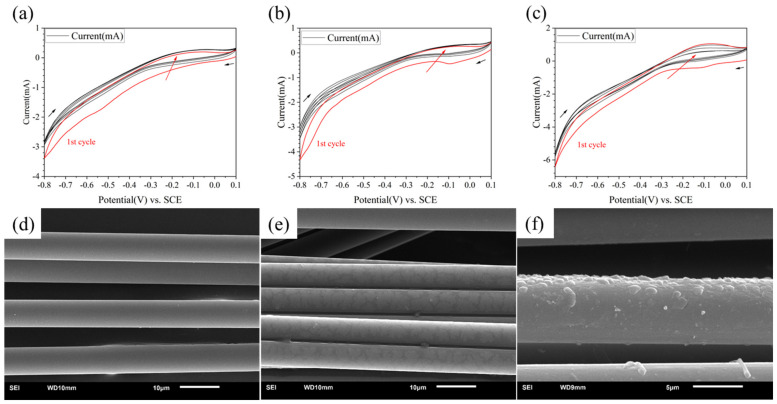
CV curves and SEM image of modified CF in different concentration of PPD (**a**,**d**) 5 mmol/L (**b**,**e**) 10 mmol/L (**c**,**f**) 20 mmol/L.

The CV curves are shown in Figure 2a–c. The first cycle curve in each group was painted red. The black arrows showed the direction of potential change and the red arrow showed the potential change as the number of cycles increased. It could be found that when the concentration of PPD was 5 mmol/L, almost no redox reaction occurred. When the concentration reached 10 mmol/L, a reaction peak was found at the potential of −0.1 V, indicating that grafting has occurred. The reaction peak almost only appeared in the first cycle. When the concentration reached 20 mmol/L, there was not only a reduction reaction, but also a certain oxidation reaction, indicating that there was also a certain degree of side reactions during the reaction process. The side reaction referred to the self-polymerization of a series of small molecules in PPD in the solution.

From the results of the SEM (see in Figure 2d–f), when the concentration was 5 mmol/L, there was almost no change in the surface; when the concentration was 10 mmol/L, a discontinuous thin layer appeared on the CF surface; when the concentration was 20 mmol/L, the resin layer on the CF surface became thicker. The degree of bulge completely covered the fiber layer itself, which indicated that the reactant concentration was too high. Therefore, setting the reactant concentration between 5 mmol/L and 20 mmol/L was more conducive to finding the optimal reaction concentration.

Therefore, the CF could be uniformly modified with a reactant concentration below 20 mmol/L. Side reaction will also happen with higher concentrations of PPD.

### 3.2. Effect of Different Molecules on Modified CF/PEEK Interface

**Figure 3:**
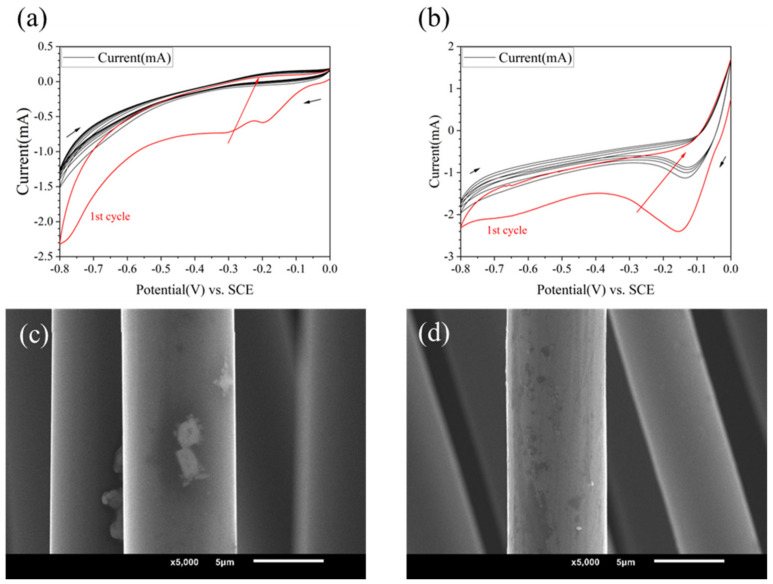
CV curves and SEM image of modified CF in different kinds of molecules (**a**,**c**) 10 mmol/L ABA (**b**,**d**) 10 mmol/L ASA.

Using different grafting molecules as variables, when the electrolyte was 10 mM, and PPD, ABA and ASA were used as grafting agents, respectively, the CV curves are shown in Figure 2b and Figure 3a,b. It could be found that the reactions all occurred successfully. PPD, ABA, and ASA produced reduction peaks at potentials of −0.11 V, −0.25 V, and −0.17 V, respectively. ASA and ABA had higher reduction reaction activity than PPD, resulting in more obvious reduction. However, from the SEM images (see in Figure 3c,d), the morphology changes on modified CF were not significant.

The elemental compositions on the surface of different modified CF were tested by XPS; see in Table 2. The results showed that, compared with the controlled group (HCl), after grafting with ABA, ASA, and PPD, the characteristic functional group elements had changed significantly. For example, in the ASA group, the proportion of sulfur increased by 0.9%, in the PPD group, the proportion of nitrogen increased by 1.1%, and in ABA, ASA group oxygen content increased; the increases were 0.8% and 1.9%, respectively. This further demonstrated that the grafting reaction successfully occurred on the CF surface.

**Figure 4:**
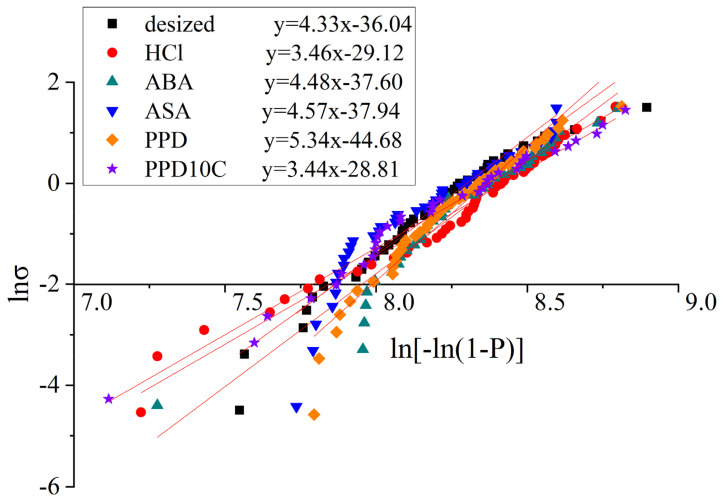
Fitting curves of the single fiber tensile test with different modified CF.

The fitting curves of the single fiber tensile test with different modified CF was shown in Figure 4 and the fitting results are shown in Table 3. The standard error was noted in Table 4. In Table 4, n was the number of samples. SE, A was the standard error of slope, which equals to m in Equation (4), while SE, B was the standard error of slope and intercept, which equals to $-mln\sigma_{0}$ in Equation (4). The Kolmogorov–Smirnov (K-S) test was also conducted. The significance level of the test was set as 5%. The K-S statistic D_n_ and critical value D_(n,0.05)_ are shown in Table 4. It was found that D_n_ < D_(n,0.05)_ in all kinds of samples, which means the data had a 95% significance level that conformed to the Weibull distribution. The above analysis indicates that the fitting quality is good.

The results of the single fiber tensile strength showed that the single fiber tensile strength of the desized CF is 3768.75 MPa. Compared with the desized CF, the single fiber tensile strength of the CF after cyclic voltammetry treatment fluctuated between 3678.38 MPa and 4088.90 MPa. The strength of the single fibers did not decrease by more than 10% compared with desized CF, indicating that the method of this experiment had little impact on the fiber strength. Due to the lower concentration of the acid selected and the smaller current intensity, the etching effect would not be as strong as that produced by electrochemical oxidation treatment. 

**Figure 5:**
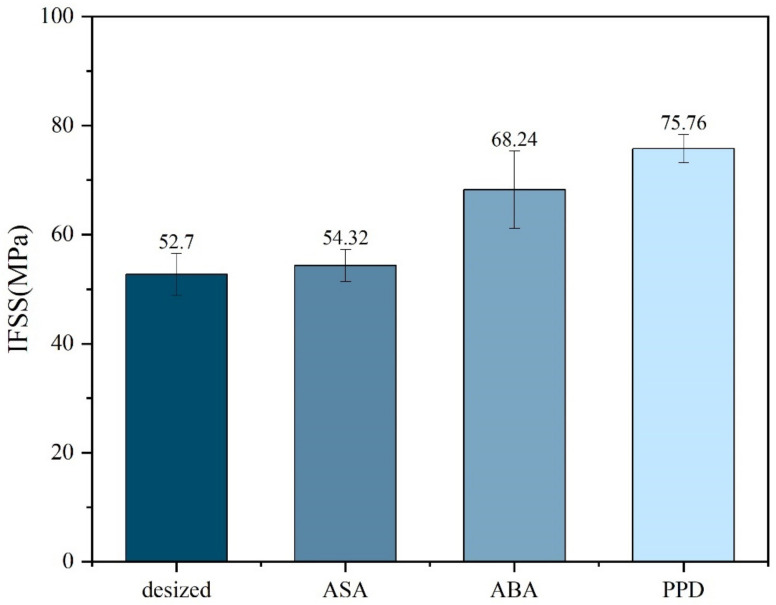
IFSS of different molecule-modified CF.

Micro-debonding test was conducted on the CF before and after modification. The results are shown in Figure 5. It could be observed from the micro-debonding test that the IFSS of the modified material had improved, among which the improvement of PPD was the most obvious, increasing from 52.70 MPa without modification to 75.76 MPa after PPD grafting. The reason for the increase in IFSS might be the increase in hydrogen bonding. Elements such as N, O and S in ASA, ABA, and PPD can generate hydrogen bonds with oxygen-containing functional groups such as carbonyl groups on PEEK, improving the adhesion between CF and PEEK. PPD, on the other hand, had a higher degree of functionalization and a relatively smaller amino group, making it easier to contact with PEEK. The amount of grafting was relatively larger, so the IFSS improvement was also greater [2,30].

### 3.3. Effect of CNTs and Different PPD Concentration on Modified CF/PEEK Interface

After comparing the three graft molecules of ABA, ASA and PPD, it could be found that PPD modification had a greater improvement in IFSS without damaging the strength of CF. Grafting small molecules is not difficult for CF, but in order to further improve the interfacial properties of CF/PEEK composites, it is necessary to utilize the effect of mechanical interlocking and improve the surface roughness of CF to further improve the interfacial properties of CF/PEEK composites. As a difunctional aniline molecule, PPD is expected to be able to react after being connected to CF to achieve connection with other components in the electrolyte, such as CNTs. The results after grafting CNTs are shown below. PPDxC represents that the concentration of PPD is x mmol/L.

**Figure 6:**
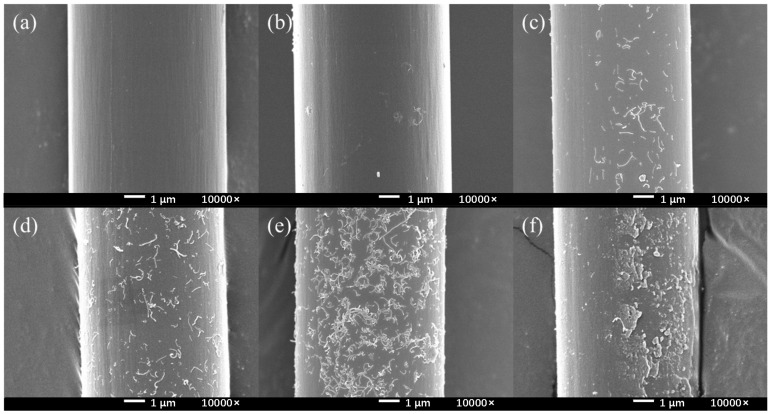
The SEM images of CNTs-modified CF in different PPD concentrations (**a**) HCl (**b**) HCl + NaNO_2_ (**c**) PPD10C (**d**) PPD20C (**e**) PPD30C (**f**) PPD40C.

According to the Figure 6, there was no change in the controlling group (HCl, HCl + NaNO_2_). In the absence of electrochemical treatment, only a small part of the CNTs were attached on the surface. Because of the post-treatment ultrasonic vibration, the attached CNTs could easily fall off from the CF surface. As PPD was added and the concentration was increased, the amount of CNTs on the CF surface gradually increased, and the CNTs could be stably retained at the CF surface. When the PPD concentration was between 10 mmol/L and 30 mmol/L, it could be observed that CNTs were attached to the surface in a very uniform state. When the PPD concentration increased, it could be observed that the grafting amount of CNTs decreases, the grooves on the CF surface gradually disappear, and more polymers are attached. When the PPD concentration reached 40 mmol/L, the CF surface was completely attached by the polymer layer, and CNTs were basically invisible on the CF surface. These phenomena showed that at higher PPD concentrations, the side reaction of mutual polymerization between PPD occurred first and hindered further reactions with CNTs. The above experiments showed that after electrochemical treatment, CNTs did not exist in the form of adsorbing on the surface, but generated chemical bonds and had a stronger interaction with CF.

**Figure 7:**
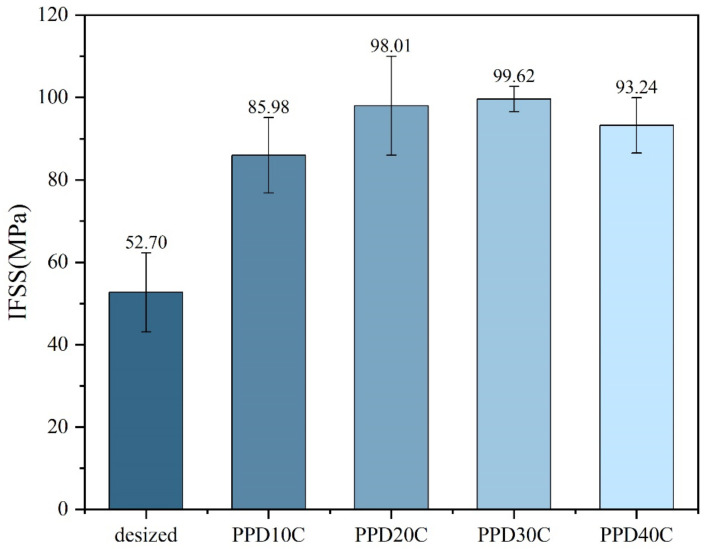
IFSS of CNTs-modified CF in different PPD concentrations.

The results of micro-debonding measurement on PPD-CNT grafted CF with different concentrations are shown in Figure 7. When CNT grafting was performed, IFSS was higher than PPD grafting at all concentrations, indicating that the introduction of CNT was beneficial to the improvement of IFSS. When the PPD concentration was around 30 mmol/L, the IFSS reached the highest level of 99.62 MPa, with an increase of 89.03%. The data of IFSS also showed the same trend as the CNT grafting amount. When the PPD concentration increased to 40 mmol/L, CNT was merely attached to CF, and the IFSS also decreased.

In order to explain the working principle of CNT and PPD on the interface, the contact angle and surface energy were tested, and the results are shown in Table 5. It could be found in the figure that as the concentration of PPD increased, the contact angle of modified CF gradually decreased, which showed that changes in PPD concentration mainly affect the fiber surface contact angle. As the concentration of PPD gradually increases, the surface energy increased as well. In addition, the polar component of surface energy was increasing along with the PPD concentration, indicating that more -NH_2_ was attached on the CF surface, which could enhance the infiltration of PEEK.

**Figure 8:**
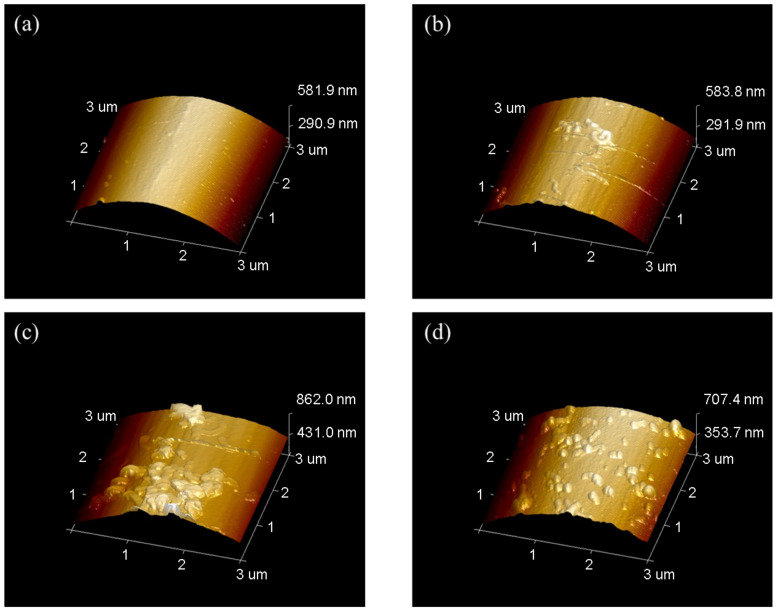
AFM images of CNTs-modified CF in different PPD concentrations (**a**) PPD10C (**b**) PPD20C (**c**) PPD30C (**d**) PPD40C.

The AFM images was shown in Figure 8, where Rq and Ra were mean-square value and arithmetic mean of roughness. As the concentration of PPD increased from 10 mmol/L to 30 mmol/L, CNTs were loaded more on the CF surface. However, when the PPD concentration reached 40 mmol/L, due to the higher degree of side reactions, more oligomer particles appeared on the CF surface, thus inhibiting further attachment of CNTs. This trend was also related to the changes of IFSS.

**Figure 9:**
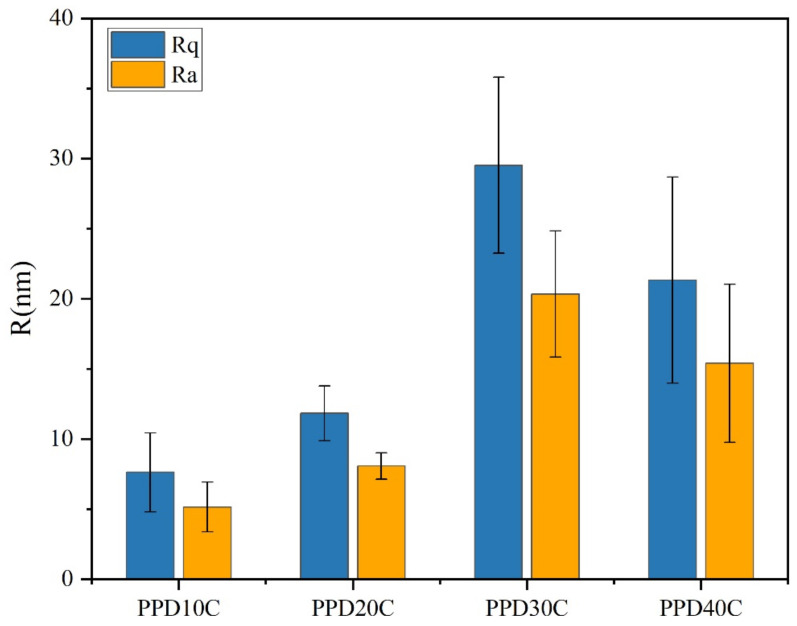
The roughness of CNTs-modified CF in different PPD concentrations.

The result of roughness was shown in Figure 9. It was observed that the PPD30C had the highest Rq or Ra. The trend of roughness changes was similar to IFSS changes. However, though the roughness of PPD40C was higher than PPD20C, the IFSS of PPD40C was still lower than PPD20C. This showed that roughness was not the only factor that determined interfacial strength. Mechanical interlocking at the interface also played a role in the stiffness of the structure that causes roughness changes. As rigid particles, CNTs could provide more PEEK pinning sites on the CF surface while stably grafting, and can exert a better interfacial modification effect.

**Figure 10:**
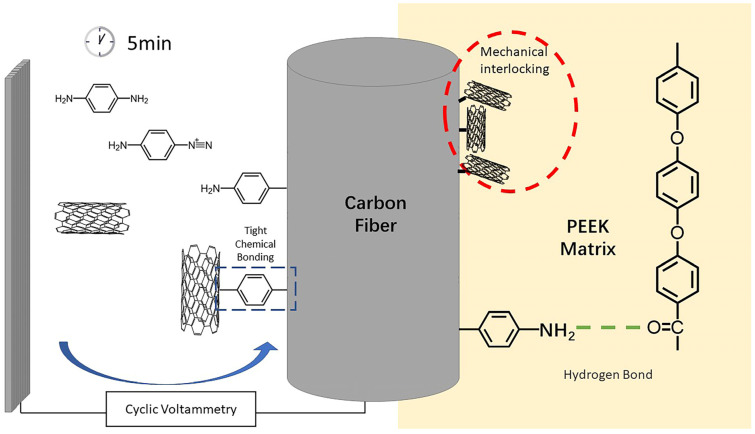
Interface enhancement mechanism of modified CF/PEEK.

Therefore, the interface enhancement mechanism of modified CF/PEEK could be summarized by two parts, which were shown in Figure 10. Firstly, the grafted PPD brought abundant amino groups onto the CF surface, allowing hydrogen bonds to form between CF and PEEK matrix. Hydrogen bonds enhanced the interaction between CF and PEEK. On the other hand, the attachment of CNTs increased the rigid rough sites on the surface of CF, thus promoting the mechanical interlocking at CF/PEEK interface. Amino groups could also improve the wettability of CF/PEEK interface, allowing more PEEK to infiltrate to the little grooves of CF.

## 4. Conclusions

In this work, the method of aryl diazonium salt reaction was conducted to modify CF/PEEK interfacial properties. Aromatic amino small molecules such as PPD, ABA and ASA were successfully grafted on the surface of CF. The single fiber tensile strength of all modified CF maintained 95% or more of desized CF. By controlling the types of reaction molecules, it was found that PPD had the highest improvement on the interfacial properties of CF/PEEK composites. When grafting in an electrolyte containing 10 mmol/L PPD, the IFSS of CF/PEEK composites increased from 52.70 MPa to 75.76 MPa, with an increase of 43.75%. The formation of hydrogen bonds between small molecule functional groups and PEEK matrix was an important reason for the improvement of interfacial performance.

By using PPD as a linking molecule through aryl diazonium salt reaction, CNTs was successfully stably loaded onto the CF, further enhancing the IFSS of CF/PEEK composite materials. The enhancement of roughness and wettability is an important reason for the improvement of IFSS. The addition of CNT further enhanced the mechanical interlocking effect and increased IFSS. There was an optimal concentration for PPD addition. When concentration of PPD was 30 mmol/L, IFSS was highest and reached 99.62 MPa, with an increase of 89.03%. If the concentration was higher than 30 mmol/L, the side effect was too strong, inhibiting the connection of CNTs.

The method of modifying CF with aromatic diazonium salts under CV conditions had minimal damage to CF, short reaction time, stable loading of CNTs, and potential for large-scale production. This method provides ideas for improving the interfacial properties of CF/PEEK composites, which is beneficial for the further application of CF/PEEK composites.

## Figures and Tables

**Table 1:** The surface tension of testing liquids for contact angle and surface energy.

Liquid	$\gamma_{l}$ (mJ·m^−2^)	$\gamma_{l}^{p}$ (mJ·m^−2^)	$\gamma_{l}^{d}$ (mJ·m^−2^)
water	72.80	51.00	21.80
ethylene glycol	47.70	16.80	30.90

**Table 2:** The elemental compositions on the surface of modified CF.

	Atomic (%)			
	C 1s	O 1s	N 1s	S 2p
HCl	82.51	15.46	1.92	0.11
ABA	81.57	16.29	2.02	0.12
ASA	79.06	17.33	2.54	1.07
PPD	80.48	16.11	3.20	0.21

**Table 3:** The result of single fiber tensile test on modified CF.

Sample Name	m	$\sigma_{0}$	$\bar{\sigma }$
desized	4.33	4139.19	3768.75
HCl	3.46	4547.45	4088.90
ABA	4.48	4445.86	4055.95
ASA	4.57	4027.12	3678.38
PPD	5.34	4281.28	3945.97
PPD10C	3.44	4323.51	3886.60

**Table 4:** The result of single fiber tensile test on modified CF.

Sample Name	n	SE, A	SE, B	D_n_	D_(n,0.05)_
desized	45	0.1501	1.2348	0.0941	0.2027
HCl	47	0.0860	0.7107	0.0984	0.1984
ABA	41	0.1880	1.5562	0.1501	0.2124
ASA	42	0.2602	2.1280	0.1323	0.2099
PPD	49	0.1976	1.6315	0.0925	0.1943
PPD10C	40	0.0881	0.7251	0.1084	0.2150

**Table 5:** Contact angle and surface energy of CNTs-modified CF in different PPD concentrations.

Sample	θ Water (°)	θ Ethylene Glycol (°)	$\gamma_{s}^{p}$ (mJ·m^−2^)	$\gamma_{s}^{d}$ (mJ·m^−2^)	$\gamma_{s}$ (mJ·m^−2^)
PPD10C	87.67	63.26	5.70	19.89	25.59
PPD20C	82.10	60.93	9.90	16.45	26.35
PPD30C	79.49	58.04	11.25	16.72	27.97
PPD40C	62.59	45.62	26.70	12.12	38.82

## Data Availability

The data presented in this study are available upon request from the corresponding author. The data are not publicly available due to the fact that they are also part of an ongoing study.
